# Which psychological needs profile exhibits higher engagement and favorable attitudes toward interprofessional education? A cluster analysis among health and social care Hong Kong students

**DOI:** 10.1186/s12909-024-06507-7

**Published:** 2024-12-20

**Authors:** John Ian Wilzon T. Dizon, Qing He, Xiaoai Shen, Pauline Luk, Doris Yin Kei Chong, Karen Man Kei Chan, Chad Wing Nga Chan, Sarah So Ching Chan, Jacky Chak Pui Choy, Chor Yin Lam, Diana Pui Ling Lee, Michael Magtoto Manio, Zoe Lai Han Ng, Terry Tin Wai Ng, Mine Orlu, Cecilia Tin Yan Sit, Grace Pui Yuk Szeto, Jacqueline Kwan Yuk Yuen, George L. Tipoe, Fraide A. Ganotice

**Affiliations:** 1 Bau Institute of Medical and Health Sciences Education, Li Ka Shing Faculty of Medicine, The University of Hong Kong, Hong Kong SAR, China; 2 Department of Physiotherapy, School of Nursing and Health Studies, Hong Kong Metropolitan University, Hong Kong SAR, China; 3 Swallowing Research Laboratory, Faculty of Education, The University of Hong Kong, Hong Kong SAR, China; 4 Department of Social Work and Social Administration, Faculty of Social Sciences, The University of Hong Kong, Hong Kong SAR, China; 5 Department of Orthopaedics and Traumatology, Li Ka Shing Faculty of Medicine, the University of Hong Kong, Hong Kong SAR, China; 6 Academic Unit of Human Communication, Learning and Development, Faculty of Education, The University of Hong Kong, Hong Kong SAR, China; 7 School of Biomedical Sciences, Li Ka Shing Faculty of Medicine, The University of Hong Kong, Hong Kong SAR, China; 8 School of Nursing, Li Ka Shing Faculty of Medicine, The University of Hong Kong, Hong Kong SAR, China; 9 School of Pharmacy, University College London, London, UK; 10 School of Medical and Health Sciences, Tung Wah College, Hong Kong SAR, China; 11 Department of Medicine, School of Clinical Medicine, Li Ka Shing Faculty of Medicine, The University of Hong Kong, Hong Kong SAR, China

**Keywords:** Basic psychological needs, Cluster analysis, Person-centered analysis, Interprofessional education, Student engagement

## Abstract

**Background:**

This study aimed to investigate which basic psychological needs profile, based on different levels of autonomy, competence, and relatedness, could exhibit higher student engagement and favorable attitudes toward interprofessional education (IPE).

**Methods:**

A total of 341 undergraduate and postgraduate health and social care students enrolled in an IPE simulation participated in this study. Data were analyzed using a person-centered approach using a two-step cluster analysis, multiple analysis of variance, and bootstrapped independent t-tests. The participants completed the self-report scales such as the basic psychological needs in general questionnaire, interprofessional attitudes scale, and engagement versus disaffection with learning scale.

**Results:**

Two basic psychological needs profiles emerged from the cluster analysis: a high basic psychological needs profile (i.e., high autonomy, moderately high competence, and very high relatedness) and a low basic psychological needs profile (i.e., low autonomy, moderately low competence, and very low relatedness). Students with high basic psychological needs profiles (*n* = 140; 41%) had more positive attitudes about IPE and were more behaviorally and emotionally engaged in participating in IPE tasks than students with low basic psychological needs profiles (*n* = 201; 59%).

**Conclusions:**

Findings suggest that health and social care students’ engagement and attitudes toward interprofessional education differed based on their basic psychological needs profiles. Health professions educators can leverage students’ basic psychological needs in designing interventions and simulation activities to promote students’ engagement and collaborative outcomes in IPE. Enhancing students’ basic psychological needs could be crucial in fostering greater behavioral and emotional engagement and positive attitudes in participating in IPE.

**Supplementary Information:**

The online version contains supplementary material available at 10.1186/s12909-024-06507-7.

## Introduction

Interprofessional education (IPE) has gained significant recognition in health and social care education curricula as it advocates for collaboration among professionals, rather than competition, to transform collaborative practice by breaking down educational silos [1]. Recent systematic reviews and meta-analytic studies show that IPE has a positive impact on the improvement of the quality of coordinated patient-centered care [2] and the knowledge and collaborative competencies of healthcare students [3–5]. As researchers explore the mechanisms behind these positive outcomes [6–8], increasing research attention has focused on understanding the relationship between students’ motivation and interprofessional collaboration outcomes [9, 10], including attitudes toward interprofessional learning [11] and engagement [12, 13].

While previous studies have established that satisfying basic psychological needs (BPN; i.e., autonomy, competence, relatedness) is related to favorable student interprofessional attitudes [11] and greater engagement [10, 12–14], significant research gaps remain. Most notably, limited research exists on how students’ engagement and attitudes toward IPE might be explained by their unique psychological needs profiles. In addition, despite the rich knowledge generated through variable-centered approaches, which focus on average motivation levels [15, 16], person-centered approaches [17] that identify distinct configurations of BPN remain unexplored in the context of IPE.

This present study aims to address these research gaps by examining the potential differences in students’ engagement and attitudes toward IPE based on the different levels of their BPN via a person-centered approach. This investigation advances the IPE and healthcare education literature in three important ways. *Theoretically*, by examining students’ profiles as a whole, we can gain a more comprehensive understanding of their basic psychological makeup. This understanding may reveal how their psychological characteristics may impact their engagement and attitudes toward interprofessional learning in healthcare education. *Methodologically*, this study utilized a person-centered approach to investigate BPN profiles among health and social care students. These profiles, characterized by different levels of autonomy, competence, and relatedness, were examined to determine which configuration would yield the most favorable attitudes and engagement in IPE simulation. *Practically*, the findings could provide medical educators and IPE program implementers valuable insights into the psychological processes underlying students’ motivation and engagement. These insights could inform tailored interventions to optimize interprofessional attitudes and learning outcomes. Below, we highlight the theory and prior studies relevant to our study and expound on the research gaps that exist within the current literature.

### Self-determination theory, basic psychological needs, and IPE outcomes

Self-determination theory (SDT), a macro theory of human motivation and functioning, posits that all people have inherent and universal basic psychological needs [18]. These needs—autonomy, competence, and relatedness—are “innate psychological nutriments that are essential for ongoing psychological growth, integrity, and well-being” (p.229) [18]. SDT further suggests that the fulfillment of these BPNs leads to optimal functioning. Due to these assertions, SDT and BPN have been examined and applied across a wide range of contexts including education [19], healthcare education [20–22], medical education [1, 23], and interprofessional education [10, 11, 13].

In IPE, SDT is employed as a theoretical framework linking basic psychological needs (BPN) to interprofessional collaboration outcomes such as attitudes toward IPE and student engagement. Attitudes toward IPE refer to students’ perceptions of interprofessional competencies, which encompass values/ethics, roles/responsibilities, interprofessional communication, and teams/teamwork [24]. These competencies are often used as indicators of IPE intervention success and are commonly examined as outcome variables by previous studies [11, 25–27]. On the other hand, student engagement, another emerging topic in IPE, involves behavioral engagement (effort, attention, persistence) and emotional engagement (enthusiasm, interest, enjoyment) [28]. Owing to its proximal and positive predictive relationship with academic achievement [29, 30], engagement has become a key outcome variable in medical and health professions education [31] and interprofessional education studies [10, 13, 14].

Previous findings suggest that after an interprofessional simulation, pre-licensure healthcare students’ sense of autonomy, competence, and relatedness predicted increased collaboration (team effectiveness and collective dedication) and behavioral (engagement and goal achievement) outcomes [10]. Another study involving medical and nursing students showed that perceived competence was related to higher autonomous motivation and improved interprofessional attitudes and competencies scores at least one year after the IPE intervention [11]. Further, a study among healthcare education students has found that higher levels of autonomy, competence, and relatedness are positively related to behavioral engagement [12]. Overall, these studies highlight that SDT is a viable theoretical framework that can explain how BPN influences attitudes and engagement in IPE. However, some potential research gaps seem to remain.

### Research gaps

While research on both engagement and attitudes toward IPE and their links with basic psychological needs is increasing, there was less focus on how students’ engagement and attitudes toward IPE might be explained by their unique psychological needs profiles. Examining how combinations of basic psychological needs could influence students’ engagement and attitudes toward IPE is important to further unpack the established link between motivation and IPE outcomes [9–13].

In addition, as previously mentioned, past studies have examined how basic psychological needs (BPN) are associated with IPE outcomes such as team effectiveness, goal achievement, engagement [10, 13], and IPE competencies and attitudes [11] using regression-based analyses and ANOVAs. However, although these analytical methods were appropriate and fit for the aims of these previous studies, it is also meaningful to employ statistical analyses that examine subgroups or profiles of students in IPE based on different combinations of autonomy, competence, and relatedness needs can provide insights into how these needs influence engagement and attitudes toward IPE. Additionally, those previous studies used variable-centered approaches [15, 16], which may not fully account for distinct patterns of BPN levels among individuals. Utilizing a data-driven approach to explore potential differences in engagement and attitudes toward IPE based on students’ BPN may uncover important patterns or profiles.

Cluster analysis [32] allows researchers to utilize a person-centered approach in analyzing the data to identify subgroups or profiles based on patterns in the scores among the study variables. Instead of restricting predetermined cut-off scores, cluster analysis allows the data to naturally group the participants following their similarities in the study variables [33]. For example, we can use cluster analysis to identify BPN profiles of students based on the different levels of their needs for autonomy, competence, and relatedness. Lastly, with the identified profiles or clusters, researchers can compare outcomes or variables of interest based on these profiles. Cluster analysis has also been used in previous studies in medical education [34], in healthcare education [35], and in interprofessional education [36].

### The present study

In summary, previous studies have investigated BPNs and their association with outcomes in IPE [10, 11, 13] and explained the findings using the SDT as a theoretical framework. However, no study has yet examined how students’ engagement and interprofessional attitudes (i.e., IPE outcomes) could differ based on the levels of their basic psychological needs. Further, previous studies analyzed their data using variable-centered approaches, regression-based analyses, and analyses of variance (ANOVA).

Therefore, using a person-centered approach, and guided by SDT as a theoretical framework, the aim of this study is to extend our current understanding of how basic psychological needs profiles could influence engagement and attitudes toward IPE. We hypothesize that:

H_1_: Students with profiles that exhibit high autonomy, high competence, and high relatedness will exhibit the best favorable attitudes toward interprofessional learning; and.

H_2_: Students with profiles that exhibit high autonomy, high competence, and high relatedness will also exhibit greater behavioral and emotional engagement.

## Methods

### Participants, study design, and procedures

Of the 395 total undergraduate and postgraduate health and social care students enrolled in an interprofessional education (IPE) simulation course, 341 voluntarily consented to participate in this study (86.33% response rate; *n* = 54/13.67% were excluded for not meeting the inclusion criteria; see Supplementary Fig. 1). The inclusion criteria include that the participants must be undergraduate students, are enrolled in IPE simulation courses led by the designated university from January to February 2023, provided voluntary informed consent to participate in the study, and completed the survey. The exclusion criteria include non-undergraduate students, students who are on leave of absence during the data collection period, students who are unenrolled in the IPE simulation courses, those who did not provide their informed consent to participate, and those who have incomplete survey data. The final participants were from Medicine (*n* = 67), Nursing (Bachelor’s programme; *n* = 155), Nursing (Master’s programme; *n* = 12), Physiotherapy (Bachelor’s programme; *n* = 36), Social Work (Bachelor’s programme; *n* = 9), Social Work (Master’s programme; *n* = 30), and Speech and Hearing Sciences (Bachelor’s programme; *n* = 32). The mean age of participants was 22.11 years old, ranging from 19 to 28 years old. Most participants were female (*n* = 228; 66.86%). Supplementary Table 1 shows further details about the demographic characteristics of the participants.

This quantitative cross-sectional study was conducted during the two three-week IPE simulation courses implemented from January to February 2023 at the University of Hong Kong. After participating in each IPE simulation course, an online survey link using Qualtrics was provided to the students. The two IPE simulation courses focused on a *dementia* case (in January 2023) and a *fracture* case (in February 2023), respectively. The design and sequence of activities for both the IPE simulation courses can be found in Supplementary Fig. 2. Informed consent was sought from the participants at the beginning of each survey. Their consent or non-consent to voluntarily participate had no bearing on their academic assessment for the simulation courses. Lastly, the ethics and procedures of the study were approved by the University of Hong Kong’s Human Research Ethics Committee for Non-clinical Faculties (approval number EA220544).

### Measures

#### Demographic characteristics

We have collected the demographic characteristics of the participants by asking them about their age, gender, discipline, and year level.

#### Basic psychological needs

We used the 21-item Basic Psychological Need Satisfaction Scale in General (BPNSS-General) [18, 37] to measure the participants’ competence, autonomy, and relatedness needs. The self-report scale’s items were slightly tweaked to adapt to the context of IPE. For example, the original item from the competence subscale (Cronbach’s a = 0.55) “*People I know tell me I am good at what I do*” was adapted to “*People in my IPE team tell me that I am good at what I do*”. Another example is the adaptation of the original item from the autonomy subscale (Cronbach’s a = 0.71) “*I generally feel free to express my ideas and opinions*” to “*I generally feel free to express my ideas and opinions in my IPE team*”. A final example is the original item from the relatedness subscale (Cronbach’s a = 0.79) “*People in my life care about me*” adapted to “*People in my IPE team care about me*”. Participants responded to each item using the Likert scale from 1 (Not true at all) to 7 (Very true). Higher mean scores for each subscale indicate higher need satisfaction for that specific basic psychological need. This adapted scale was also previously validated among Asian interprofessional undergraduate students [10].

#### Interprofessional attitudes

We used the 27-item Interprofessional Attitudes Scale (IPAS) [38] to measure the participants’ attitudes about interprofessional education and collaborative practice. This self-report scale has five subscales, namely: Teamwork roles and responsibilities (e.g., “*Shared learning before graduation will help me become a better team worker*”; Cronbach’s a = 0.91), Patient-Centeredness (e.g., “*Establishing trust with my patients is important to me*”; Cronbach’s a = 0.94), Interprofessional Biases (e.g., “*I have prejudices or make assumptions about health professionals/students from other disciplines*”; Cronbach’s a = 0.75), Diversity and Ethics (e.g., *“Understand what it takes to effectively communicate across cultures*”; Cronbach’s a = 0.92), and Community-Centeredness (e.g., “*Work on projects to promote community and public health*”; Cronbach’s a = 0.95). Participants responded to the items using the Likert scale ranging from 1 (Strongly disagree) to 5 (Strongly agree). This scale has also been validated among Asian interprofessional undergraduate students [39].

#### Behavioral and emotional engagement

We used five items from the behavioral engagement subscale (Cronbach’s a = 0.91) and five items from the emotional engagement subscale (Cronbach’s a = 0.89) of the 20-item Engagement vs. Disaffection with Learning scale (EVDL) [28]. We adapted the self-report scale’s items to fit the IPE context. For example, the original item from the behavioral engagement subscale “*I try hard to do well in school*” was adapted to “*I try hard to do well in IPE*” and the original item from the emotional engagement subscale “*When I’m in class*, *I feel good*” was adapted to “*When I’m in IPE*, *I feel good*”. Participants responded to each item using the Likert scale ranging from 1 (Not at all true) to 4 (Very true).

### Data analysis

As no missing data were found, all data from the final 341 participants were used in the analyses. We used the Statistical Package for the Social Sciences (SPSS version 28) [40] in all of our data analyses. Descriptive statistics such as mean, standard deviation, skewness, and kurtosis were yielded from the data using the *Analyze* and *Descriptive* functions in SPSS. We also mean standardized the variables autonomy, competence, and relatedness. Given the current study’s exploratory nature, theoretical alignment (i.e., SDT’s proposition that the three BPN operate together rather than separately), and comparable sample size with previous studies that employed clustering approaches [41, 42], we used the two-step clustering approach. The two-step clustering approach is suitable for handling continuous data simultaneously while maintaining statistical robustness [43]. First, we used a hierarchical cluster analysis using Ward’s method and Squared Euclidean distance to extract the number of clusters from the data. Second, after we identified two clusters based on the resulting dendrogram, we ran a K-means cluster analysis to yield the final cluster centers and to classify the participants based on their cluster membership.

Using the yielded two cluster profiles, we ran an independent t-test with 1,000 bootstrap samples and examined whether there would be significant differences in the behavioral and emotional engagement and interprofessional attitudes of the participants belonging to either of the two cluster profiles. A *p*-value less than 0.05 was considered significant. We also computed Cohen’s *d* as a measure of effect size with values of 0.2 (small effect size), 0.5 (medium effect size), and 0.8 (large effect size) as reference points of interpretation [44].

## Results

### Descriptive statistics and basic psychological needs profiles

**Fig. 1:**
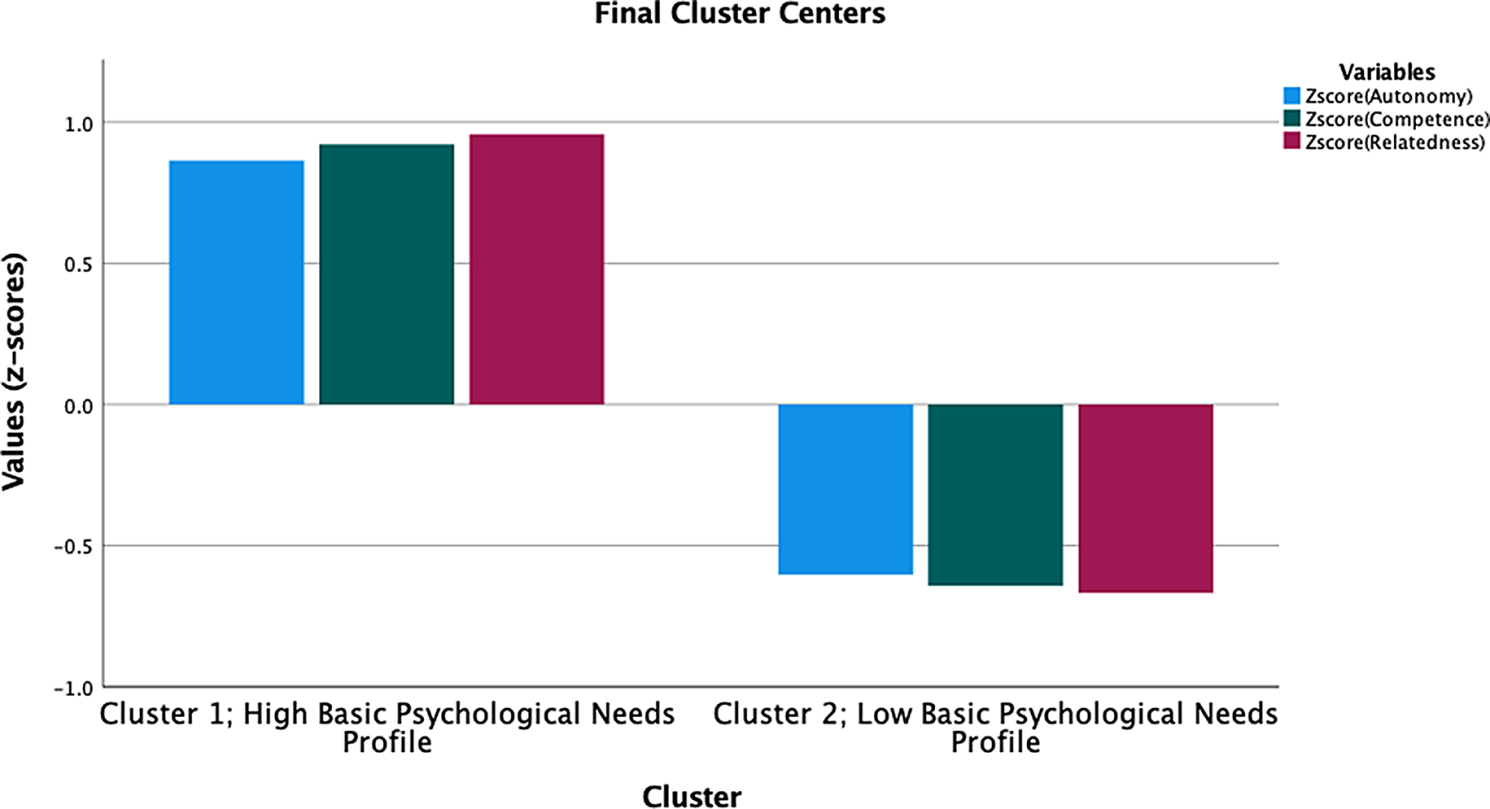
The extracted basic psychological needs profiles. Notes: *Cluster 1*: High basic psychological needs profile (i.e., high autonomy, moderately high competence, and very high relatedness) and *Cluster 2*: Low basic psychological needs profile (i.e., low autonomy, moderately low competence, and very low relatedness); X-axis represents the two basic psychological needs profiles. Y-axis represents z-scores for the three basic psychological needs

Supplementary Table 2 shows the descriptive statistics results for each of the study variables. Two basic psychological needs profiles were yielded from the data (see Fig. 1): A high basic psychological needs profile is characterized by high autonomy (z = 0.86), moderately high competence (z = 0.92), and very high relatedness (z = 0.96) and a low basic psychological needs profile that represents low autonomy (z = -0.60), moderately low competence (z = -0.64), and very low relatedness (z = -0.67). 41% (*n* = 140) of the participants were classified as having high psychological needs profiles while 59% (*n* = 201) were classified as having low basic psychological needs profiles.

In addition, Table 1 shows the distribution of the two basic psychological needs profiles across the disciplines. Results showed that the highest proportion of students with high basic psychological needs profiles were from Physiotherapy (52%) while the highest proportion of students with low basic psychological needs profiles were from Social Work (Bachelor’s programme; 75%). Lastly, no gender differences were observed from the basic psychological needs profiles as there was a higher proportion of low basic psychological needs profiles in both females and males (see also Table 1).

**Table 1:** Composition of extracted basic psychological needs clusters based on Discipline and Gender

	Cluster 1: High basic psychological needs group (*n* = 140; 41%)	Cluster 2: Low basic psychological needs group (*n* = 201; 59%)	Total
*Discipline*			
1. Medicine	30 (45%)	37 (55%)	67 (100%)
2. Nursing – Bachelors	59 (38%)	96 (62%)	155 (100%)
3. Nursing – Masters	5 (42%)	7 (58%)	12 (100%)
4. Physiotherapy	17 (52%)	16 (48%)	33 (100%)
5. Social Work – Bachelors	3 (25%)	9 (75%)	12 (100%)
6. Social Work – Masters	11 (37%)	19 (63%)	30 (100%)
7. Speech and Hearing	15 (47%)	17 (53%)	32 (100%)
**Total**	140 (41%)	201 (59%)	341 (100%)
*Gender*			
Female	101 (44.30%)	127 (55.70%)	228 (100%)
Male	41 (36.28%)	72 (63.72%)	113 (100%)
**Total**	142 (41.64%)	199 (58.36%)	341 (100%)

### Differences in interprofessional attitudes between high and low basic psychological needs profiles

**Fig. 2:**
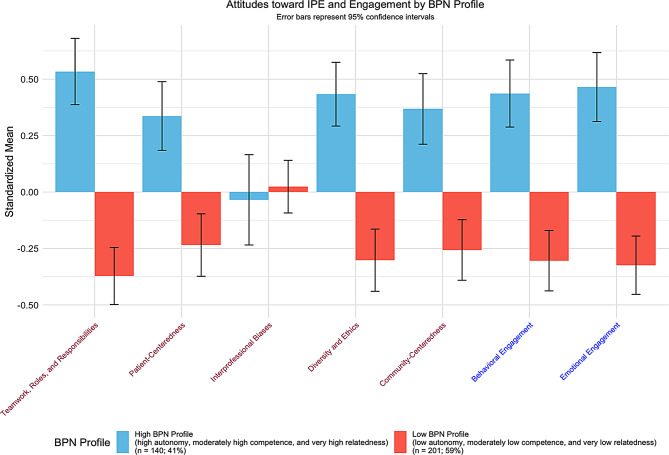
Attitudes toward interprofessional education and engagement subscales mean scores differ based on the two basic psychological needs profiles. Notes: BPN = Basic Psychological Needs. All attitudes toward IPE subscales’ means significantly differed between high vs. low BPN profiles except for “Interprofessional Biases”. Both engagement subscales’ means significantly differed between high vs. low BPN profiles

Based on the independent t-test results (Table 2), we found that there were significant differences among the students who have high and low BPN profiles in terms of their interprofessional attitudes. More specifically, we found that students who have high BPN profiles had higher mean scores across all the interprofessional attitudes subscales than those with low basic psychological needs profiles (except for the Interprofessional Biases subscale). Students with high basic psychological needs profiles (BPNP) had higher mean scores in the teamwork, roles, and responsibilities subscale [High BPNP: 4.42 (0.55) vs. Low BPNP: 3.86 (0.57), *t*(339) = 9.18, *p* < .001; *d* = 0.67], patient-centeredness subscale [High BPNP: *M* = 4.46 (SD = 0.57) vs. Low BPNP: *M* = 4.10 (SD = 0.57), *t*(339) = 5.40, *p* < .001; *d* = 0.60], diversity and ethics subscale [High BPNP: *M* = 4.50 (SD = 0.55) vs. Low BPNP: *M* = 4.02 (SD = 0.64), *t*(339) = 7.16, *p* < .001; *d* = 0.79], and community-centeredness subscale [High BPNP: *M* = 4.40 (SD = 0.59) vs. Low BPNP: *M* = 4.40 (SD = 0.59), *t*(339) = 5.96, *p* < .001; *d* = 0.66] (See Fig. 2).

**Table 2:** Mean scores on interprofessional attitudes across the two basic psychological needs profiles

Outcomes	Cluster 1 (*n* = 140, 41%)		Cluster 2 (*n* = 201, 59%)		t		df		*p*		Bootstrapped 95% C.I. Lower		Bootstrapped 95% C.I. Lower		Cohen’s d		Cluster comparison	
	M	SD	M	SD														
1. TRR	**4.42**	0.55	3.86	0.57		9.18		339		< 0.001	0.23	0.46		0.67		1 > 2		
2. PC	**4.46**	0.57	4.10	0.62		5.40		339		< 0.001	0.23	0.49		0.60		1 > 2		
3. IB	3.50	1.11	3.55	0.77		-0.53		339		0.298	-0.25	0.15		-0.06		ns		
4. DE	**4.50**	0.55	4.02	0.64		7.16		339		< 0.001	0.35	0.61		0.79		1 > 2		
5. CC	**4.40**	0.59	4.00	0.61		5.96		339		< 0.001	0.26	0.52		0.66		1 > 2		

*Notes*: TRR = Teamwork roles and responsibilities; PC = Patient-centredness; IB = Interprofessional biases; DE = Diversity and ethics; CC = Community-centredness. Bolded values are the highest mean scores and are statistically significant at *p* < .001. ‘ns’ = not significant. ‘*df*’ = degrees of freedom. Cluster 1 = High basic psychological needs group and Cluster 2 = Low basic psychological needs group. Bootstrap results were based on 1,000 bootstrap samples

### Differences in behavioral and emotional engagement between high and low basic psychological needs profiles

Table 3 shows the independent t-test results that compared the behavioral and emotional engagement mean scores of the students that belonged to either the high or low basic psychological needs clusters. We found that students who have high basic psychological needs profiles (BPNP) had higher mean scores in both behavioral [High BPNP: *M* = 3.58 (SD = 0.45) vs. Low BPNP: *M* = 3.20 (SD = 0.49), *t*(339) = 7.21, *p* < .001; *d* = 0.79] and emotional engagement [High BPNP: *M* = 3.38 (SD = 0.50) vs. Low BPNP: *M* = 2.95 (SD = 0.51), *t*(339) = 7.77, *p* < .001; *d* = 0.85] than those with low BPNP (see also Fig. 2).

**Table 3:** Mean scores on behavioural and emotional engagement across the two basic psychological needs profiles

Outcomes	Cluster 1 (*n* = 140, 41%)		Cluster 2 (*n* = 201, 59%)		t		df		*p*		Bootstrapped 95% C.I. Lower		Bootstrapped 95% C.I. Upper	Cohen’s d	Cluster comparison	
	M	SD	M	SD												
1. Behavioural engagement	**3.58**	0.45	3.20	0.49		7.21		339		< 0.001		0.28	0.48	0.79	1 > 2	
2. Emotional engagement	**3.38**	0.50	2.95	0.51		7.77		339		< 0.001		0.32	0.54	0.85	1 > 2	

*Notes*: Bolded values are the highest mean scores and are statistically significant at *p* < .001. ‘ns’ = not significant. ‘*df*’= degrees of freedom. Cluster 1 = High basic psychological needs group and Cluster 2 = Low basic psychological needs group. Bootstrap results were based on 1,000 bootstrap samples

## Discussion

This study examined which basic psychological needs (BPN) profile exhibits favorable interprofessional attitudes and greater behavioral and emotional engagement among interprofessional undergraduate and postgraduate students. We hypothesized that students with profiles that exhibit high autonomy, competence, and relatedness will exhibit favorable attitudes toward interprofessional learning (H_1_) and greater behavioral and emotional engagement (H_2_). Our results support both of our hypotheses.

### Basic psychological needs profiles of health and social care students

The findings revealed two distinct profiles: a high BPN profile (high autonomy, moderately high competence, and very high relatedness) and a low basic psychological needs profile (low autonomy, moderately low competence, and very low relatedness). Generally, a higher proportion of students (59%) exhibited a low basic psychological needs profile. This higher proportion of low BPN profiles was also observed in both females and males. Previous findings have noted that university students in the East (e.g., China) tend to have lower mean basic psychological needs compared to students from Western countries (e.g., Belgium, USA, Peru) [45]. Such findings may also be similar to the participants in the current study (i.e., who are predominantly Hong Kong Chinese students) and may be further explained by cultural differences [46].

On the other hand, in terms of the disciplines involved in the study, Physiotherapy had the highest proportion of students with high BPN profiles while Social Work (Bachelor’s programme) had the highest proportion of students with low BPN profiles. These findings may be explained by the difference in the number of participants per discipline, by program-specific or discipline-specific factors, or even student-level differences that may influence how both cohorts of students fulfill or inadvertently undermine their autonomy, competence, and relatedness needs.

### Differences in attitudes toward IPE and engagement: high vs. low basic psychological needs profiles

Our findings demonstrated significant differences in interprofessional attitudes (H_1_) and engagement (H_2_) between the two profiles. Students with a high basic psychological needs (BPN) profile reported more favorable attitudes toward interprofessional teamwork, roles, responsibilities, patient-centeredness, diversity, ethics, and community-centeredness, aligning with previous research on the importance of fulfilling BPN for fostering positive interprofessional attitudes [11]. Of note, we found no significant differences between the two BPN profiles and students’ interprofessional biases. This finding may mean that regardless of their BPN profiles, IPE students tend to have more or less similar perceptions about having prejudices or assumptions of students from other disciplines than themselves. On the other hand, students with a high BPN profile exhibited higher levels of behavioral and emotional engagement in interprofessional learning activities, corroborating the link between need satisfaction and engagement explored by previous studies [10, 13]. These findings underscore the important role of autonomy, competence, and relatedness in shaping students’ attitudes and engagement within interprofessional education contexts.

Based on SDT [18], fulfillment of the three BPNs is crucial for optimal functioning and motivation. In the IPE context, these BPNs are recognized as predictors of collaboration outcomes such as engagement, goal achievement, and collective dedication, among others [10]. In addition, these BPNs also promote positive attitudes toward IPE [11]. In our study findings, we extended these findings as we highlight the influence of the different levels of autonomy, competence, and relatedness on student engagement and attitudes toward IPE. More specifically, we found that students with high BPN profiles had favorable attitudes toward IPE and greater behavioral and emotional engagement. Conversely, we also found that students with low BPN profiles had less favorable attitudes toward IPE and lesser behavioral and emotional engagement.

In the study’s IPE context, the activities (e.g., team readiness assurance test, designing interprofessional healthcare plans) were designed to provide students choice, voice, and ownership in directing their learning progress, promoting autonomy needs fulfillment. These activities were similar to the activities designed and used in previous studies in IPE [9, 13]. Students from different disciplines were randomly assigned to teams, bringing their disciplinary knowledge, and fostering competence needs fulfillment. Given IPE’s collaborative nature [1], facilitated and unfacilitated group discussions fostered group cohesion, connectedness, and relatedness, fulfilling relatedness needs. These design elements could plausibly explain the different autonomy, competence, and relatedness levels observed. Findings from the current study highlight that enhancing students’ fulfillment of the three BPNs is also crucial in the IPE context. Teaching strategies such as establishing connections with students from other disciplines, providing room for autonomy in accomplishing the IPE activities, and designing activities with increasing levels of difficulty are potential ways to enhance students’ relatedness, autonomy, and competence needs, respectively, in IPE. Given that the context of IPE is social by nature, educators and IPE program implementers may consider designing further interventions primarily targeting relatedness needs fulfillment, followed by competence and autonomy needs.

### Study limitations and future recommendations

Despite the notable findings of the present study, we also note some limitations. First, the study used a cross-sectional research design, which captured the students’ BPN, engagement, and attitudes toward IPE at a single time point. Although such a design is appropriate for the aim of the study, changes among the variables across time could not be examined accordingly. Future studies could explore the same variables across multiple time points (i.e., longitudinally) to compare temporal changes among students’ BPN profiles and IPE outcomes. Second, our data were based on self-report measures completed by the students. Although such measures were psychometrically sound and were previously used in the medical, healthcare, and interprofessional education contexts, self-report measures are also known to introduce some level of response bias and social desirability bias. Future studies may consider assessing students’ attitudes toward IPE and engagement using tutors/teachers’ observations to triangulate students’ self-reports. In addition, future researchers can consider including a social desirability scale to filter participants’ responses for potential social desirability bias. Lastly, although the sample size in the present study is comparable to previous studies that investigated the same study variables (e.g., Ganotice et al. [10]), a larger sample size with a more balanced number of students per discipline is also ideal to further stabilize the resulting proportions from the cluster analysis and to increase the generalizability of future findings.

## Conclusion

This study employed cluster analysis to examine how health and social care students’ behavioral and emotional engagement, and attitudes toward interprofessional education (IPE) may differ based on their basic psychological needs (BPN) profile (high vs. low autonomy, competence, and relatedness). Students with a high BPN profile exhibited more positive interprofessional attitudes and greater engagement compared to those with a low BPN profile. The study extends previous findings on the BPN-outcomes link in IPE using a person-centered approach. The findings can aid educators and program implementers in improving students’ BPNs and collaborative experiences, and in promoting positive interprofessional attitudes and engagement. We hope that this study contributes to the ongoing debate in understanding how to promote engagement and favorable attitudes toward interprofessional learning among students and inspire future research in this area.

## Electronic Supplementary Material

Below is the link to the electronic supplementary material.

Supplementary Material 1

Supplementary Material 2

Supplementary Material 3

Supplementary Material 4

## Data Availability

The data that support the findings of this study can be requested from the corresponding author, F.A.G., upon reasonable request.

## Abbreviations

IPE: Interprofessional Education
BPN: Basic Psychological Needs
SDT: Self-Determination Theory
ANOVA: Analysis of Variance
BPNSS: Basic Psychological Needs Satisfaction Scale
IPAS: Interprofessional Attitudes Scale
EVDL: Engagement versus Disaffection with Learning
SPSS: Statistical Package for the Social Sciences
BPNP: Basic Psychological Needs Profile
