# Supplementary material for: Which psychological needs profile exhibits higher engagement and favorable attitudes toward interprofessional education? A cluster analysis among health and social care Hong Kong students
Source: BMC Med Educ. 2024 Dec 20;24:1508. doi: 10.1186/s12909-024-06507-7 (PMC11662416; doi:10.1186/s12909-024-06507-7)
Supplement: Supplementary file 1 — Supplementary Material 1 [file 12909_2024_6507_MOESM1_ESM.docx]

*Supplementary Table 1*. Demographic characteristics

| Characteristic | *n* | % |
| --- | --- | --- |
| *Gender* |  |  |
| Female | 228 | 66.86 |
| Male | 113 | 33.14 |
| *Discipline* |  |  |
| Medicine | 67 | 19.65 |
| Nursing - Bachelors | 155 | 45.45 |
| Nursing - Masters | 12 | 3.52 |
| Physiotherapy | 36 | 10.56 |
| Social Work - Bachelors | 9 | 2.64 |
| Social Work- Masters | 30 | 8.80 |
| Speech and Hearing Sciences | 32 | 9.38 |
| *Year level* |  |  |
| Year 2 | 90 | 26.4 |
| Year 3 | 36 | 10.6 |
| Year 4 | 140 | 41.1 |
| Year 5 | 32 | 9.4 |
| Year 1 Masters | 31 | 9.0 |
| Year 2 Masters | 12 | 3.5 |

*Note. n = 341.* Participants were on average 22.11 years old (*SD* = 2.62). The age range is 19 to 28 years old.
