# Supplementary material for: Which psychological needs profile exhibits higher engagement and favorable attitudes toward interprofessional education? A cluster analysis among health and social care Hong Kong students
Source: BMC Med Educ. 2024 Dec 20;24:1508. doi: 10.1186/s12909-024-06507-7 (PMC11662416; doi:10.1186/s12909-024-06507-7)
Supplement: Supplementary file 2 — Supplementary Material 2 [file 12909_2024_6507_MOESM2_ESM.docx]

*Supplementary Table 2***.** Descriptive statistics of the study variables

| Variable | Mean | SD | Kurtosis | Skewness |
| --- | --- | --- | --- | --- |
| 1. Autonomy | 4.71 | 0.72 | 0.44 | 0.54 |
| 2. Competence | 4.94 | 0.92 | -0.21 | 0.07 |
| 3. Relatedness | 5.03 | 0.92 | -0.62 | 0.15 |
| 4. Teamwork roles and responsibilities | 3.92 | 0.55 | 2.17 | -0.84 |
| 5. Patient-centeredness | 4.25 | 0.62 | 2.63 | -1.04 |
| 6. Interprofessional biases | 3.53 | 0.92 | -0.32 | -0.39 |
| 7. Diversity and ethics | 4.22 | 0.65 | 1.53 | -0.89 |
| 8. Community-centeredness | 4.17 | 0.63 | 2.20 | -0.85 |
| 8. Behavioral engagement | 3.35 | 0.51 | -0.74 | -0.13 |
| 9. Emotional engagement | 3.13 | 0.55 | -0.14 | -0.08 |
