# Supplementary material for: Which psychological needs profile exhibits higher engagement and favorable attitudes toward interprofessional education? A cluster analysis among health and social care Hong Kong students
Source: BMC Med Educ. 2024 Dec 20;24:1508. doi: 10.1186/s12909-024-06507-7 (PMC11662416; doi:10.1186/s12909-024-06507-7)
Supplement: Supplementary file 3 — Supplementary Material 3 [file 12909_2024_6507_MOESM3_ESM.docx]

**Supplementary Figure**

*Supplementary Figure 2.* The IPE simulation course model implemented at the [redacted for peer review] in 2023.

**
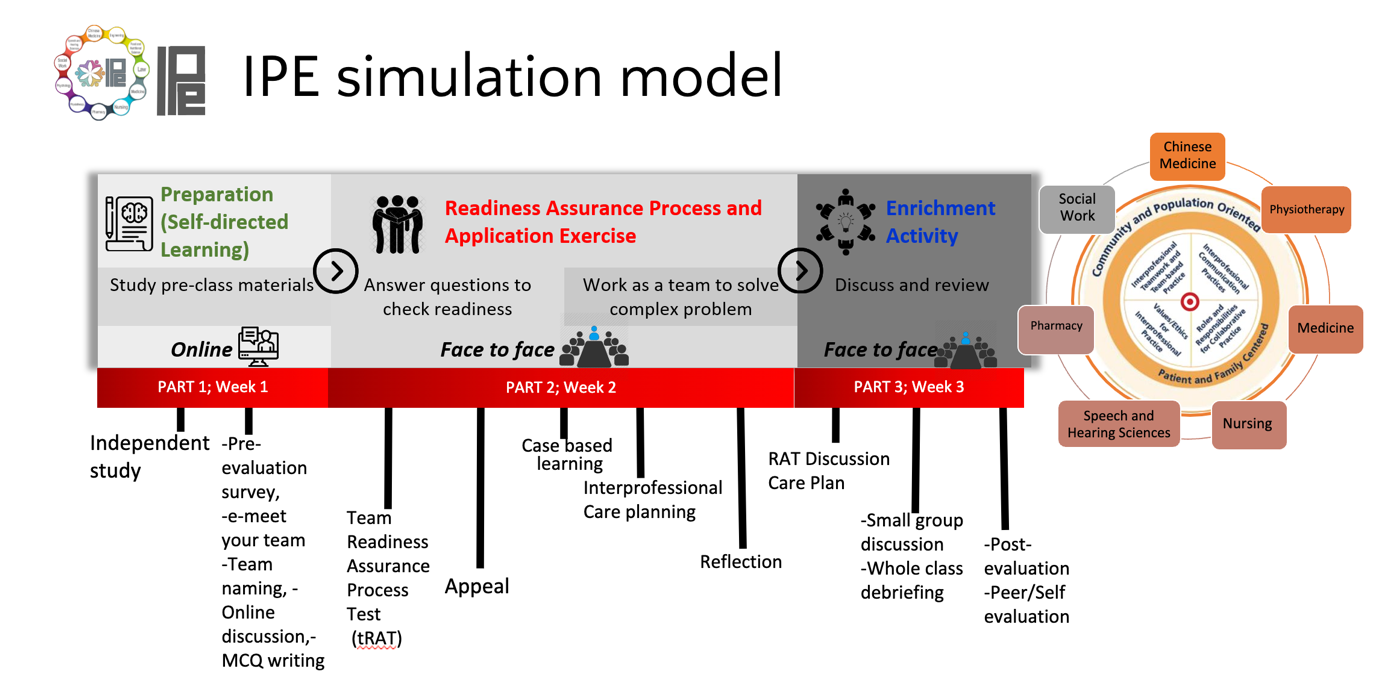
**

Notes: MCQ; Multiple Choice Questions; RAT = Readiness Assurance Test
