# Supplementary material for: Which psychological needs profile exhibits higher engagement and favorable attitudes toward interprofessional education? A cluster analysis among health and social care Hong Kong students
Source: BMC Med Educ. 2024 Dec 20;24:1508. doi: 10.1186/s12909-024-06507-7 (PMC11662416; doi:10.1186/s12909-024-06507-7)
Supplement: Supplementary file 4 — Supplementary Material 4 [file 12909_2024_6507_MOESM4_ESM.docx]

**Supplementary Figure**

*Supplementary Figure 1.* Participants’ recruitment flowchart

## Eligibility

Assessed for eligibility
(n = 395)

*Note*: This is the total number of enrolled students in the two IPE simulation courses

- Medicine (n = 76)
- Nursing-Bachelors (n = 181)
- Nursing-Masters (n = 12)
- Physiotherapy (n = 44)
- Social Work-Bachelors (n = 14)
- Social Work-Masters (n = 32)
- Speech and Hearing Sciences (n = 36)

Excluded
(n = 54)

*Note*: Did not consent to participate

- Medicine (n = 9)
- Nursing-Bachelors (n = 26)
- Nursing-Masters (n = 0)
- Physiotherapy (n = 8)
- Social Work-Bachelors (n = 5)
- Social Work-Masters (n = 2)
- Speech and Hearing Sciences (n = 4)

## Inclusion

Included in the study according to the inclusion criteria
(n = 341)

- E.g., with informed consent, no missing data, etc.
- Medicine (n = 67)
- Nursing-Bachelors (n = 155)
- Nursing-Masters (n = 12)
- Physiotherapy (n = 36)
- Social Work-Bachelors (n = 9)
- Social Work-Masters (n = 30)
- Speech and Hearing Sciences (n = 32)

Analyzed
(n = 341)

## Analysis
